# Harnessing Entropic Effects from Interlayer Coupling to Modulate Ion Transport and Rectification in Multilayered Janus Graphene Nanopores

**DOI:** 10.1021/jacs.5c17242

**Published:** 2026-01-09

**Authors:** Shuang Li, Xinke Zhang, Xuewei Dong, Xin You, Bing Yuan, Kai Yang

**Affiliations:** † Center for Soft Condensed Matter Physics and Interdisciplinary Research & School of Physical Science and Technology, 12582 Soochow University, Suzhou 215006 Jiangsu, China; ‡ Songshan Lake Materials Laboratory, Dongguan 523808 Guangdong, China

## Abstract

Ion transport through nanoscale channels enables advanced functionalities, such as ionic current rectification (ICR), with promising applications in neuromorphic computing and biomimetic signal processing. However, the fundamental mechanisms controlling the ion dynamics under nanoconfinement remain poorly understood. Using atomistic molecular dynamics simulations and free energy calculations, we demonstrate that multilayered Janus graphene oxide nanopores exhibit exceptional and tunable ICR performance mediated by interlayer coupling. These structures achieve a rectification ratio enhancement of up to 2 orders of magnitude—from ∼2 in a single layer to over 2000 at 3.5 V/nm in multilayered configurations—and a shift of the peak rectification field from 0.7 to 3.5 V/nm with increasing layer number. Ion distribution analyses reveal distinctive ionic enrichment-depletion behavior unique to multilayered architectures. Thermodynamically, we unveil that synergistic interlayer coupling fundamentally reshapes the free energy landscape, creating a highly asymmetric profile with multiple energy barriers and wells due to entropy—enthalpy competition. Importantly, entropy is identified to play a critical role in stabilizing energy wells and facilitating directional ion transport—a mechanism absent in single-layer systems. These insights provide a mechanistic basis for ion rectification and establish design principles, such as interlayer spacing or number control, for developing high-performance ionic membranes and nanofluidic devices.

## Graphical abstract

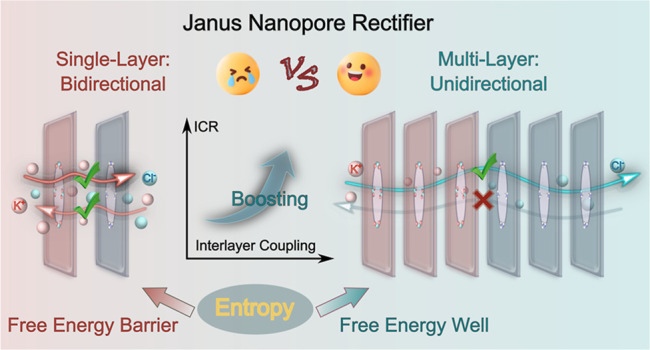

## ■ Introduction

Ion transport in nanoscale channels exhibits fundamentally distinct behavior from bulk systems, enabling advanced ionic electronic applications.^1,2^ Under nanoscale confinement, ions display distinctive dynamics that give rise to functionalities such as ionic current rectification (ICR).^3,4^ ICR originates from structural or interfacial asymmetries, which establish localized ion enrichment/depletion zones and charge gradients to control directional ion flow.^5^ This diode-like behavior serves as a cornerstone for next-generation technologies—including nanofluidic rectifiers, memristors, and biosensors—with applications in neuromorphic computing and signal modulation.^6,7^

Solid-state nanopores have thus garnered significant interest due to their structural stability and precise dimensional control.^8–10^ Among these, Janus nanopores, which feature chemically distinct bipolar surfaces, constitute a highly promising platform.^11–18^ Their inherent electrostatic asymmetry enables modulation of local ion concentrations and energy landscapes, offering the potential for achieving high rectification ratios. The ICR mechanism in Janus nanopores was elucidated by Daiguji et al. using a continuum theoretical framework.^11^ Siwy demonstrated that rectification arises from the synergy between asymmetric geometry and heterogeneous surface properties.^12^ Moreover, studies by Qiu and others show that structural and physicochemical parameters (e.g., pore length, surface charge density) effectively tune the ICR of Janus nanopores.^14–17^ Likewise, Aksimentiev et al. also showed that graphene–insulator–graphene nanopores exhibit flexible current control and rectification, offering a key basis for nanofluidic electronics.^18^ In particular, recent advances in multilayered architectures—such as stacked 2D graphene-based systems—have enabled diverse implementations of Janus-based ICR devices,^19,20^ including bipolar ionic diodes in nanochannel networks,^21^ bioinspired designs bridging semiconductor–biological interfaces,^22^ and polylysine-modified biocompatible iontronic devices.^23^ However, a key knowledge gap remains in understanding the regulatory mechanisms governing bidirectional ion transport and their influence on rectification performance. Furthermore, rational design principles for maximizing rectification efficiency remain elusive.^24,25^

On the other hand, the transport of ions through nanopores is critically governed by the intricate competition between entropy and enthalpy. It is established that both enthalpic and entropic contributions can significantly alter the height and distribution of energy barriers for ion migration.^26–30^ For instance, Qu et al. demonstrated that ion dehydration can reconfigure the entropy-enthalpy balance, which elevates the enthalpic barrier while conferring entropic gains, thereby selectively modulating the transport barriers for different anions.^26,27^ Noh et al. identified hydration entropy as the primary component of the transmembrane energy barrier for divalent cations like Mg^2+^, a mechanism that governs ion selectivity.^28^ Furthermore, Pan et al. reported that the presence of K^+^ in a nanopore can raise the energy barrier for Mg^2+^ migration by suppressing the entropy compensation associated with Mg^2+^–Cl^–^ pairing.^29^ Generally, these insights underscore that a comprehensive thermodynamic framework could offer a powerful approach to elucidating the fundamental mechanisms behind ionic current rectification in solid-state nanopores.

Herein, we performed atomistic molecular dynamics (MD) simulations and free energy calculations to systematically investigate ion transport through multilayered Janus graphene oxide (GO) nanopores. Our results showed that increasing the layer number could enhance the ICR ratio by 2 orders of magnitude and shift peak performance toward higher electric fields. This improvement arises from synergistic interlayer coupling that reshapes the free energy landscape for ion migration. Further analysis of entropy-enthalpy competition revealed the critical role of entropy in regulating the free energy landscape of ion–pore interactions and ion migration pathways in multilayered systems. These findings provide mechanistic insight into designing high-performance, rationally designed ionic rectification membranes.

## Results and Discussion

### Correlation Between Ionic Rectification Performance and Layer Number in Janus Nanopores

We constructed two types of GO sheets: COO^–^-modified (negatively charged) and NH_3_^+^-modified (positively charged), with charge densities (∼2 e/nm^2^) matching experimental values.^31^ Specifically, each 4 × 4 nm^2^ sheet featured a 1.42 × 1.26 nm^2^ quasi-circular nanopore functionalized with four edge modification groups. Representative configurations are shown in Figure 1. Mirroring experimental fabrication,^32,33^ these sheets were stacked into multilayered Janus structures with alternating charges, maintaining a default interlayer spacing (*S*) of 0.34 nm.^34,35^ Notably, while our model used idealized, perfectly aligned layers with defined spacings to clarify the underlying mechanisms, these key structural features are becoming increasingly achievable experimentally with state-of-the-art techniques.^36–38^ Herein, the systems with 1–8 layers were simulated (Figure S1), immersed in 1.0 M KCl solution (4444 water molecules +80 ions). Periodic boundary conditions, in line with previous studies,^39–41^ were applied in all three dimensions throughout the simulations.

**Figure 1:**
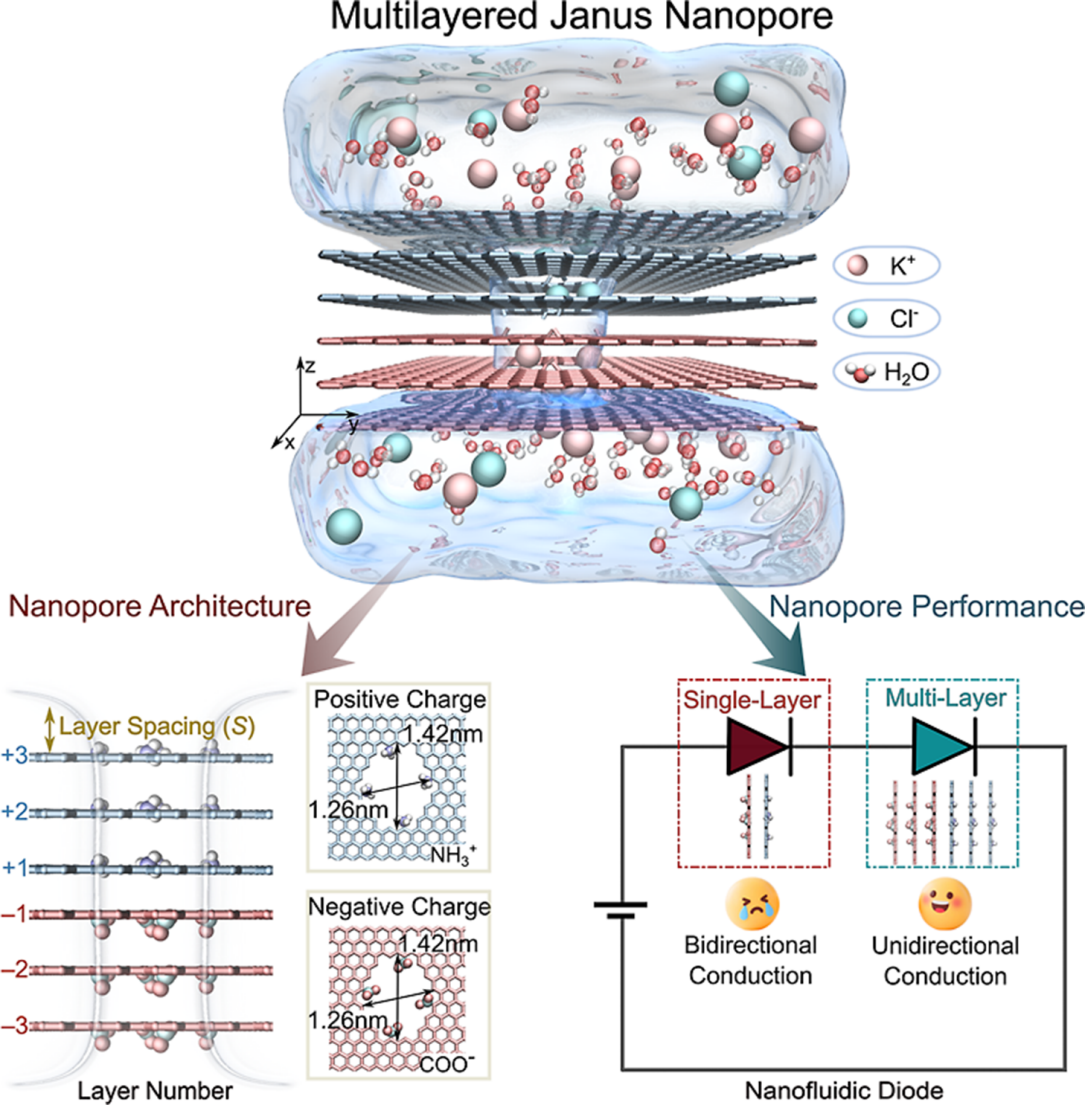
Schematic diagram of the Janus nanopore. A 3-Layer Janus graphene oxide (GO) nanopore with alternating COO^–^ (red) and NH_3_^+^ (blue) functional groups immersed in a 1.0 M KCl solution (K^+^: pink; Cl^–^: green; water molecules: red and white). The multilayered structure exhibits unidirectional current conduction, creating diode-like properties, in contrast to the bidirectional conduction observed in single-layer Janus nanopores.

To probe the ion transport dynamics, electrostatic fields (+*E*/–*E*) ranging from 0.3 to 4 V/nm were applied along the *z*-axis, exerting a global force *F* = *qE* on each charged particle.^42^ Here, ″+*E*″ and ″–*E*″ denote fields aligned with or against the Janus dipole, respectively (see Figure 1). Specifically, to capture sufficient ion transport events within practical simulation times and obtain statistically robust results, most simulations employed electric field strengths substantially higher (∼V/nm) than typical experimental values.^43–46^ Nevertheless, our control simulations indicated that the field strength has a limited influence on ion–pore interactions (e.g., on hydrated ion states; see Figure S2). Moreover, it has been shown that the simulation results obtained under these conditions remain consistent with both theoretical predictions^47^ and experimental observations.^48^

We found that simple structural modifications of these Janus nanopores, however, strongly influenced their rectification performance. Figure 2a depicts the difference in ionic current conduction between single-layer and multilayered Janus nanopores under ± *E*. Notably, when the layer number exceeds 2, the current under +*E* significantly surpasses that under –*E* (Figure 2b,c). This asymmetry stems from distinct ion-entry mechanisms: Under +*E*, ions approach the pore through an oppositely charged entrance, where electrostatic attraction partially offsets nanoconfinement effects, enhancing conduction (″ON-state″); while under –*E*, ions face a like-charged entrance, where electrostatic repulsion and spatial confinement impose a high energy barrier, inhibiting transport (″OFF-state″). Consequently, ionic fluxes under +*E* exceed those under –*E* by orders of magnitude (Figure 2b,c), demonstrating the rectification capability of multilayered Janus nanopores. Collectively, these findings reveal that the ON/OFF current ratio scales with the number of Janus layers effectively.

**Figure 2:**
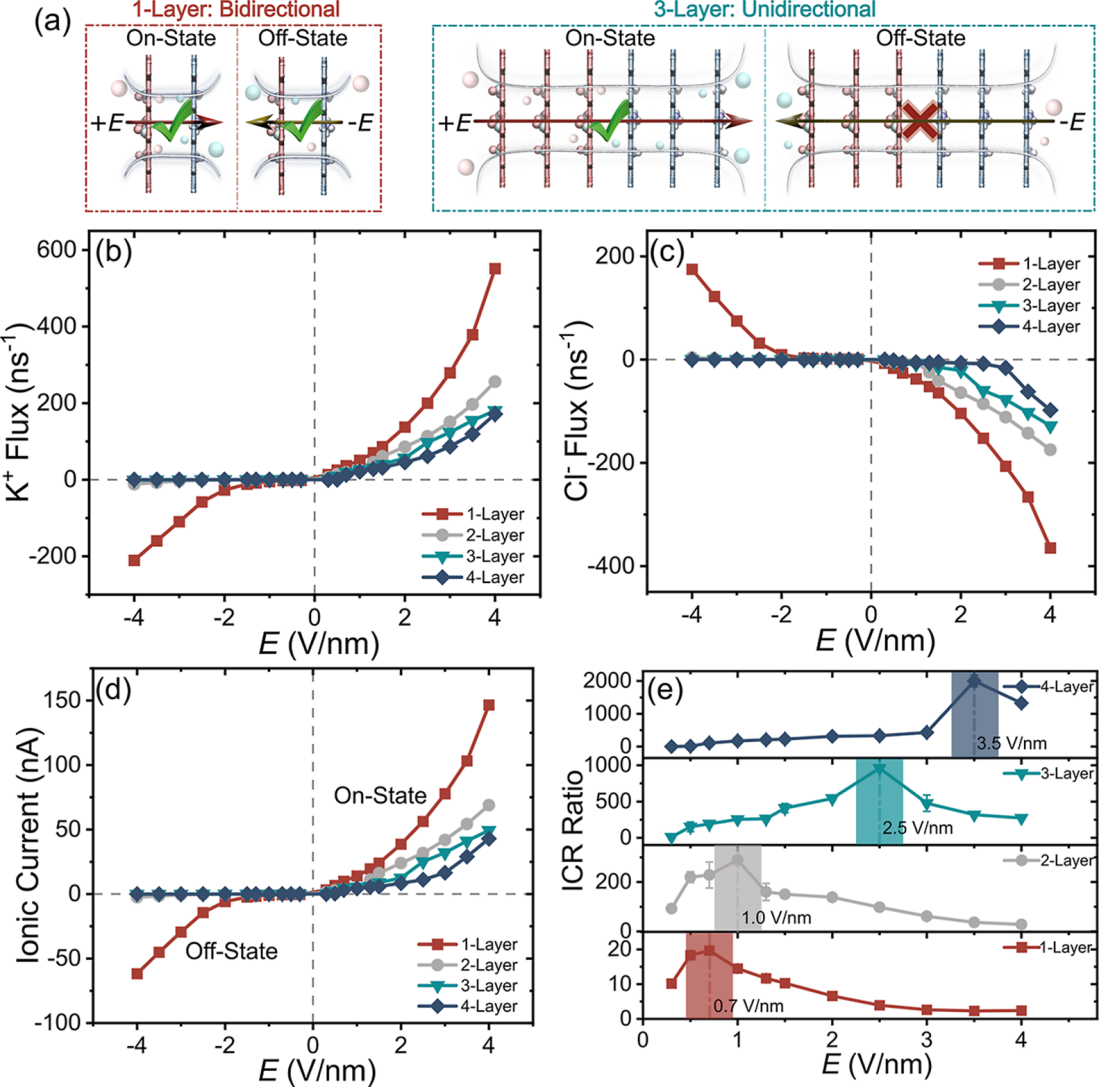
Ion transport dynamics and nanopore rectification performance. (a) Schematic diagram illustrating the difference in ionic current conduction between the 1-Layer and 3-Layer nanopores under opposing electric fields (±*E*). Flux profiles of (b) K^+^ and (c) Cl^–^, (d) ionic current, and (e) ICR ratio versus electric field *E* for distinct layer numbers. The shaded area in (e) denotes the peak ICR ratio.

Moreover, the rectification performance of Janus nanopores exhibits a strong layer number dependence characterized by three key trends. First, increasing the layer number leads to gradually decreasing ON-state ion fluxes and currents (Figure 2b–d), while OFF-state fluxes drop precipitously to near-zero levels in systems with two or more layers. Second, the ICR ratio (*I*_+*E*_/*I*_–*E*_), a crucial performance metric, increases from 10^1^ to 10^3^ as the layer number rises from one to four (Figure 2e), establishing layer modulation as an effective strategy for rectification optimization. Notably, all systems show field-dependent ICR ratios that peak at specific electric field strengths, with these optimal fields shifting to higher values as layer numbers increase (as highlighted by shaded regions in Figure 2e). Our analysis indicates that this layer-number-dependent peak shift originates from the evolving asymmetric ion distribution or ion concentration polarization (ICP).^11,23,49,50^ Specifically, as layers increase, the field required for complete depletion at –*E* rises correspondingly and aligns exactly with the ICR peak, defining the condition of maximum ionic asymmetry (see Figure S3 for details).

Furthermore, other structural properties of nanopores could affect the rectification performance. Pore size, for instance, critically influences the ICR ratio: larger pores (e.g., 2.27 × 1.73 nm^2^) reduce ICR, while excessively small pores (e.g., 0.88 × 0.79 nm^2^) severely restrict ion transport unless very high fields are applied (Figure S4). Therefore, selecting an appropriate pore size is essential for achieving high rectification. The pore shape exhibits only a minor effect on ions flux and ionic current (Figure S5). Interestingly, even in nanopores containing structural imperfections (e.g., layer misalignment), rectification behavior is still evident (Figure S6), despite a reduction in ionic current, presumably caused by an effective constriction of the pore. Taken together, these results reveal an important interplay between pore structure and applied bias, suggesting that rectification efficiency in these diode-like nanofluidic devices can be maximized through rational structural and operational parameter tuning.

In addition, while high rectification performance requires both a substantial ICR ratio and elevated ON-state current, quantitative analysis of nanopores with increasing layers (1- to 8-Layer) under representative electric fields (1.0, 2.5, and 4.0 V/nm) reveals a critical trade-off (Figure S7). Beyond 5-Layers, the OFF-state current is fully suppressed, but the ON-state current is significantly diminished. Consequently, the 3-Layer system demonstrates outstanding rectification performance, achieving both a high ICR ratio (>1000) and robust ON-state current (>32 nA) at moderate fields (e.g., 2.5 V/nm).

Generally, multilayered Janus nanopores exhibit distinct advantages in their rectification performance. This is demonstrated, for example, by their high sensitivity to pore length, where rectification can increase by up to 2 orders of magnitude as the length grows from 0.34 to 2.38 nm. Furthermore, the key ion transport behaviors are also observed in other 2D materials such as h-BN nanopores (Figure S8). These results collectively demonstrate the promising potential of multilayered Janus pores for ionic electronic applications.

### Interlayer Coupling Regulates Ion Distribution and Hydration Structure

Ion distributions within nanopores could reflect microscopic transport pathways and ion dynamics. A Janus nanopore comprises three distinct electrostatic zones: an antielectrical region, a homoelectrical region, and a central heterojunction. Consequently, ion distributions display pronounced axial/radial dependence that evolves with layer number and operational state (Figure 3a,c). In 1-Layer pores (Figure 3b), both K^+^ and Cl^–^ display low densities, indicating rapid transmembrane transport without accumulation. Conversely, multilayered systems (e.g., 3-Layer) show significant ion accumulation: under + *E* (ON-state, e.g., +1.0 V/nm), K^+^ concentrates near pore entrances (low *z*) and centralizes at exits (high *z*), while Cl^–^ exhibits the inverse pattern; this trend persists across layer configurations (Figure S9); under –*E* (OFF-state), ions accumulate exclusively in charge-complementary regions near exits (Figures 3c and S9). This nonuniform ion distribution—evident as enrichment/depletion zones—induces ICP, which is one of the primary mechanisms governing ionic rectification.^11,23,49,50^ Taken together, these distinct profiles elucidate transport asymmetry under opposing fields and demonstrate how increased layers amplify ± *E* disparities, enhancing ICR ratios.

**Figure 3:**
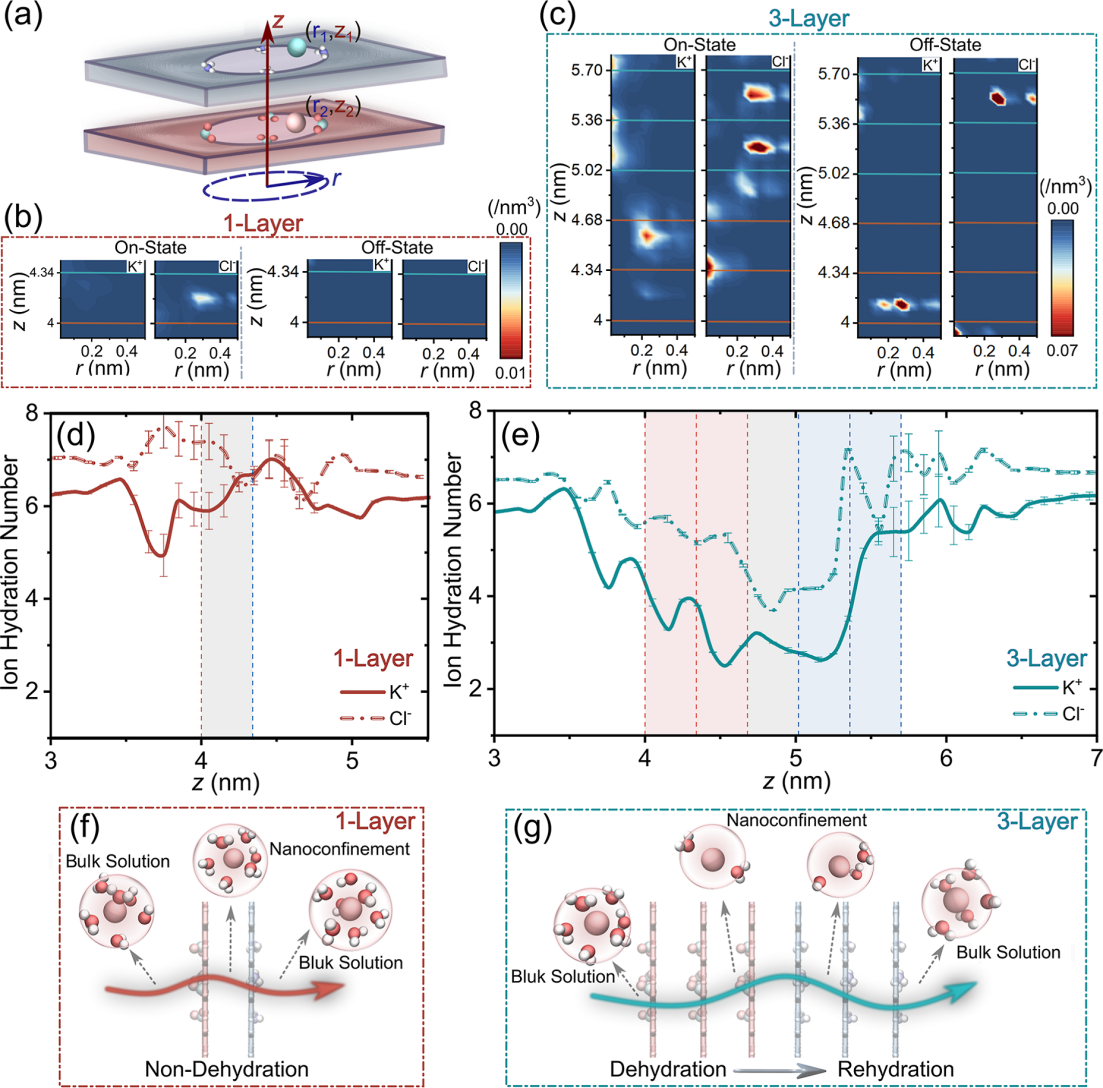
Ion density distributions and hydration dynamics in Janus nanopores. (a) Schematic diagram of ions density along the axial (*z*) and radial (*r*). (b, c) ON-state (*E* = +1.0 V/nm) and OFF-state (*E* = – 1.0 V/nm) densities for 1-/3-Layer nanopores. Horizontal lines denote COO^–^ (red)/NH_3_^+^ (blue) modification sites. (d, e) Hydration number of ions versus *z*-position at ON-state for (d) 1-Layer and (e) 3-Layer nanopores; dashed lines indicate COO^–^/NH_3_^+^ modification sites. Light red/blue regions: positive/negative charged; gray: heterojunction. (f, g) Schematic diagram of K^+^ hydration evolution during transmembrane transport for (f) 1-Layer and (g) 3-Layer nanopores at ON-state, the red/green arrows represent K^+^ transport pathways.

Also, hydrated ion size and hydration shell deformation affect ion migration through dynamic dehydration/rehydration processes.^43,51^ Analysis of axial hydration profiles across nanopores with varying layer numbers (Figures 3d,e and S10, S11) reveals distinct behaviors: during ON-state transport, 1-Layer pores exhibit minor hydration fluctuations (Figure 3d,f), whereas multilayered systems show progressive dehydration upon pore entry, reaching minima at the heterojunction before rehydrating upon exit (Figures 3e and S10). These results reveal that an increase in layer number triggers a dehydration–rehydration cycle during ion transport (as schematized in Figure 3g), and in particular, the heterojunction shows the most pronounced hydration changes, serving as the critical control point for this cyclic process. Conversely, OFF-state conditions yield negligible hydration fluctuations due to minimal ion occupancy (Figure S11). Collectively, an increase in the layer number leads to sequential dehydration–rehydration cycles that influence ion transport dynamics and efficiency in multilayered nanopores.

### Thermodynamic Mechanism of Ionic Current Rectification

Exploring the thermodynamic mechanism that governs ionic rectification in multilayered Janus nanopores—one of the central aims of this work—is helpful to clarify ion–pore interactions and ion transport behavior in nanopores. Accordingly, we computed the free energy surfaces (FESs) using on-the-fly probability enhanced sampling (OPES), with the axial (*z*) and radial (*r*) coordinates as collective variables. The calculations were performed with the PLUMED 2.9.1 plugin.^52,53^ Figures 4a,b and S12a,b present the resulting FESs, revealing the layer-dependent formation of energy barriers and wells associated with specific ion–pore interactions—including pore entry, desorption, electrostatic attraction/repulsion, and approach to the heterojunction. For K^+^ in a 1-Layer nanopore (Figure 4a,c), the FES and Δ*G* exhibit clear asymmetry: the energy increases steeply along both the *z* and *r* coordinates, culminating in a barrier of ∼ 12 kJ/mol at the heterojunction upon contact with homoelectrical residues. In contrast, Cl^–^ shows an inverse trend, with a higher energy barrier at the heterojunction (∼20 kJ/mol), consistent with its larger hydrated radius (Figure S12c). As the layer number increases, however, the FESs and Δ*G* profiles exhibit more complex changes (Figure 4b,d, and S12). While the overall asymmetry of the free energy landscape is maintained, the minimum free energy path shifts significantly due to the expansion of both high- and low-energy regions and the emergence of multiple energy barriers and wells with added layers. In contrast to the strong preference for small radial positions (*r*)—i.e., near the pore center—observed in 1-Layer pores, ions in multilayered pores follow pathways that exhibit coupled axial and radial variations. Notably, in the 3-Layer pore, interlayer coupling induces an energy well near the pore entrance and rim (*z* ∼ 4 nm, *r* ∼ 0.4 nm)—a feature absent in the 1-Layer pore. Further along *z*, additional barriers or wells arise near subsequent layers (e.g., barriers of ∼ 13.5 and 25.5 kJ/mol; Figure 4d), corresponding to ion desorption barriers. These features suggest a ″stop–go″ transport mechanism mediated by repeated adsorption–desorption cycles.^48^ As K^+^ approaches the heterojunction and the positively charged region, the FES increases markedly. Radially, steep Δ*G* gradients confine the lowest-energy path to 0 < *r* < 0.2 nm. Axially, the pronounced barrier at the heterojunction (∼22 kJ/mol) and positively charged region (∼27.5 kJ/mol) reflect the energy cost to dissociate ion pairs within the constricted nanopore.^54^ Interestingly, although Δ*G* remains generally positive throughout this region, shallow energy wells are still present near the pore exit and between layers, underscoring the distinct role of interlayer coupling. On the other hand, Cl^–^ exhibits a similar but inverted *z*-dependent FES profile, with generally higher energy barriers (Figure S12). Here, it is worth noting that the ON-state migration corresponds to decreasing *z*. Comparative analysis of energy barriers indicates that desorption and heterojunction barriers dominate in the ON-state, whereas entrance and electrostatic exclusion barriers control the OFF-state. This directional free energy asymmetry—which intensifies axially with increasing layer number—directly accounts for the observed current rectification behavior and high ICR ratios (Figure 2).

**Figure 4:**
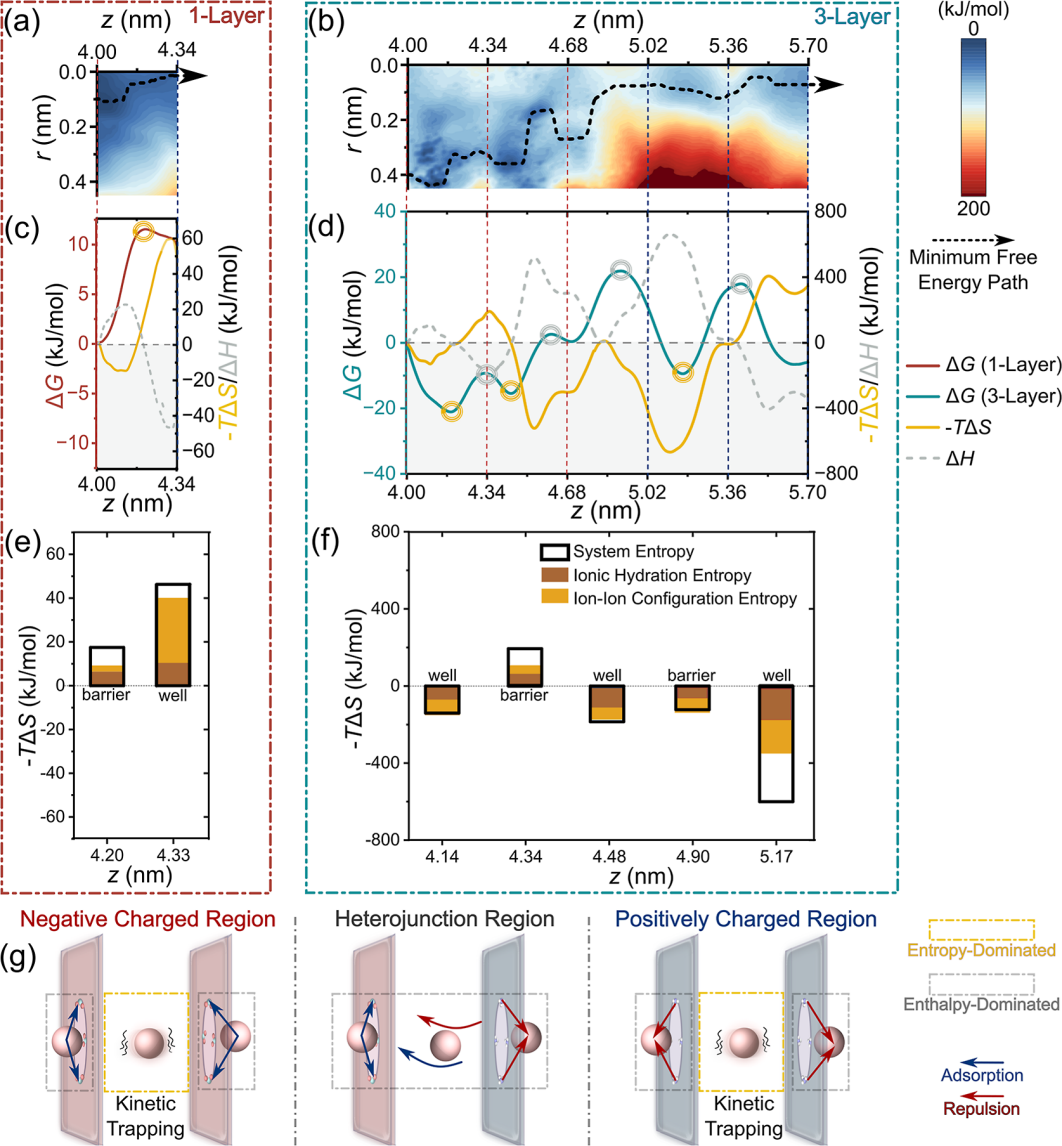
Free energy surfaces for ion transport, depicting the concomitant changes in free energy, entropy, and enthalpy along the nanopore (*z*-axis). FESs of K^+^ as a function of the axial (*z*) and radial (*r*) directions for (a) 1-Layer nanopore and (b) 3-Layer nanopore. The black dashed line represents the minimum free energy path for K^+^ transport in the ON-state. The changes in free energy (Δ*G*), entropy (Δ*S*), and enthalpy (Δ*H*) of K^+^ along the *z*-axis for (c) 1-Layer nanopore and (d) 3-Layer nanopore. The red/blue dashed lines represent the modified positions of COO^–^/NH_3_^+^. The circles in Figure 4c,d represent the peaks and valleys of the free energy barrier (yellow: entropy-dominated; gray: enthalpy-dominated). Contributions of ionic hydration entropy, and ion–ion configurational entropy to system entropy at key points along the K^+^ migration path (e) 1-Layer nanopore and (f) 3-Layer nanopore. (g) Schematic diagram of the entropy–enthalpy competition in the K^+^ transport process due to interlayer coupling in multilayered Janus nanopores.

Probing the entropy–enthalpy competition is essential to reveal the thermodynamic driving forces behind ion translocation in nanopores. Accordingly, we further quantitatively evaluated the contributions of the entropy change (Δ*S*) and enthalpy change (Δ*H*) to Δ*G*. Our results reveal a layer-number-dependent entropy–enthalpy competition in ion–pore interactions, as shown in Figure 4c,d and Table 1. In the 1-Layer nanopore, entropy (–*T*Δ*S =* 16.4 kJ/mol) primarily causes the increase in Δ*G* and the formation of the energy barrier (*z* = 4.20 nm); in contrast, in the 3-Layer system, entropy (–*T*Δ*S* < 0) makes significant contributions to stabilize energy wells and thus facilitates ion migration along the nanopore. As noted above, the variation in Δ*G* for K^+^—including the positions of energy barriers and wells—depends on the locations of the GO sheets, resulting from interlayer coupling. Specifically, energy barriers consistently occur near charged sheets, while wells are typically situated between layers (except within the heterojunction region). Strikingly, the energy barriers are predominantly enthalpy-dominated (gray circles), whereas most wells are primarily entropy-regulated (yellow circles). These observations provide mechanistic insight into ion translocation: near charged sheets, ions undergo strong electrostatic interactions (e.g., adsorption or repulsion), resulting in enthalpy-dominated barriers; conversely, kinetically trapped ions between GO sheets lead to entropy-regulated wells, as illustrated in Figure 4g. A similar entropy–enthalpy competition governs Cl^–^ transport (Figure S12c,d). Notably, this thermodynamic mechanism remains applicable to nanopores with varying sizes and shapes (Figures S4 and S5), structural imperfections (Figure S6), and even extended to other 2D material systems such as h-BN nanopores (Figure S8), which demonstrates the robust role of entropy in stabilizing ion transport across multilayered Janus nanopores.

**Table 1:** Δ*G*, Δ*H*, and *–T*Δ*S* Values for K^+^ Transport at Key Pore Positions^a^

parameters	1-Layer nanopore		3-Layer nanopore						
position (nm)	4.20	4.33	4.14	4.34	4.48	4.61	4.90	5.17	5.41
Feature	barrier	well	well	barrier	well	barrier	barrier	well	barrier
Δ*G* (kJ/mol)	11.4	10.5	–21.0	–9.3	–13.4	2.7	21.9	–9.6	18.0
–*T*Δ*S* (kJ/mol)	16.4	59.0	–140.4	193.7	–185.7	–340.0	–122.8	–601.0	43.6
Δ*H* (kJ/mol)	–5.4	–48.5	119.4	–203.5	172.2	342.7	144.7	591.4	–25.6

a All values are calculated relative to the pore entrance.

In addition, deciphering the entropy contributions arising from specific ion transport behaviors could provide deeper physical insight into the factors governing rectification. For example, our simulations show that ion hydration states change markedly during transport, particularly within multilayered pores (Figure 3). Quantitative analysis indicates that the associated entropy change constitutes a major component of the total entropy change (Figure 4e,f), thereby modulating ion–pore interactions. Furthermore, ICP stems from asymmetric ion distributions, which were also observed in our Janus nanopore systems (Figures 3 and S3). Thermodynamically, this redistribution alters the configurational entropy of the ions (Figure 4e,f), which significantly contributes to the total entropy change and consequently influences ion transport. Overall, these results underscore the critical role of interlayer coupling in modulating the entropy–enthalpy competition, and particularly highlight the entropy-mediated effects in regulating ion translocation through multilayered nanopores.

The thermodynamic picture emerging from our free energy calculations and entropy–enthalpy competition relations provides a mechanistic basis for understanding of our simulation results and offers valuable guidelines for designing advanced ionic rectification devices. For example, our free energy calculations indicate that an increase in the layer number enhances interlayer coupling, thereby amplifying free energy asymmetry and dynamic disparities between opposite ion transport directions. This coupling also introduces sequential energy barriers that collectively hinder ion conduction. In these Janus nanopores, ion migration is constrained by spatial confinement and consecutive charged sites, which thus could be regarded as a metastable process, basically even under an electric field. Together with the driving force imposed by the electric field, these factors critically influence ICR performance: In few-layer systems, low electric field strengths preserve considerable ionic current disparity between ± *E*, whereas high fields diminish the effective difference between forward and backward energy barriers—resulting in maximum ICR ratios at lower fields (e.g., 0.7 V/nm for the 1-Layer system); in contrast, multilayered systems exhibit strongly suppressed currents in both directions under low fields due to their multi barrier free energy landscapes. At high fields, however, ON-state currents are markedly enhanced with limited effect on OFF-state conduction, thereby shifting the peak ICR to higher field strengths (e.g., 1.0 V/nm for 2-Layer, 2.5 V/nm for 3-Layer, and 3.5 V/nm for 4-Layer systems). These mechanistic insights into ion dynamics in multilayered nanopores provide a valuable foundation for the rational design of high-efficiency ionic rectification devices.

### Rectification Modulation through Interlayer Coupling

Furthermore, our results indicate that interlayer coupling—and especially the associated entropic effects—play a critical role in regulating ion translocation through Janus nanopores. To further investigate this effect, we evaluated the dependence of ionic current on interlayer spacing (S = 0.24 to 1.02 nm) in the 3-Layer system as shown in Figure 5a. It is found that the ON-state current decreased sharply with increasing spacing, dropping to approximately 0.2 nA at 1.02 nm, while the OFF-state current remained negligible (Figure 5b). On one hand, this suppression is attributed to the enhanced trapping of water molecules and ions between layers at larger spacings, which hinders ionic transport (Figures S13 and S14). On the other hand, increasing the interlayer spacing—or equivalently, reducing the effective charge density—leads to higher, more separated energy barriers for ion migration (Figure 5d). The resulting weakening of interlayer coupling consequently alters key ion–pore interactions (e.g., ion hydration, Figure 5e) and overall ion transport behaviors. Quantitative analysis revealed a pronounced degradation of the ICR with increasing spacing (Figure 5c). In detail, the system with 0.68 nm spacing exhibited a one-order-of-magnitude reduction in ICR compared to the 0.34 nm reference, while rectification was nearly abolished at 1.02 nm (ICR ≈1, Figure 5c). These findings underscore the essential role of tight interlayer spacing in maintaining strong interlayer coupling and high rectification performance in multilayered Janus nanopores, providing useful design guidelines for optimized membrane materials.^55,56^

**Figure 5:**
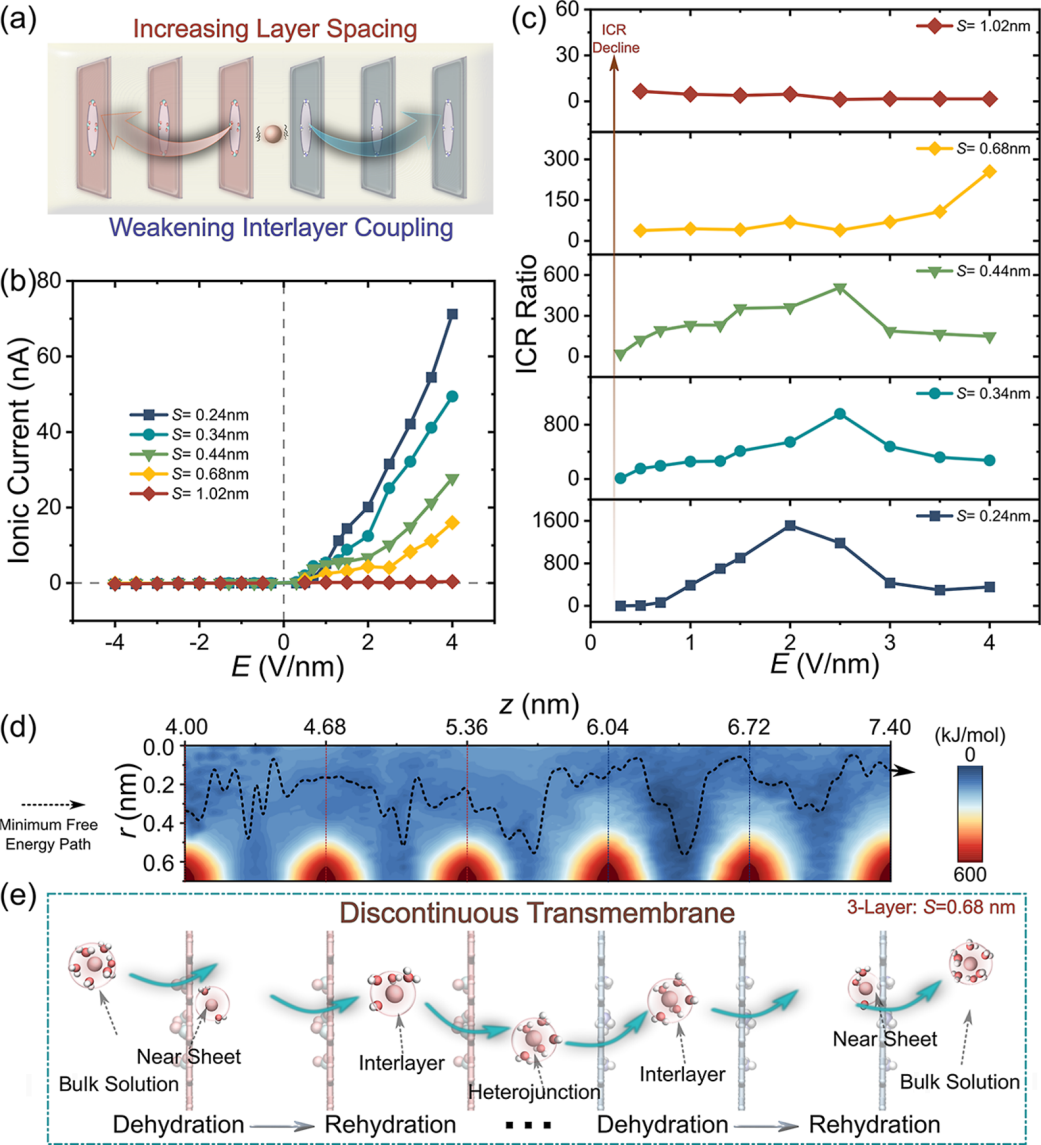
Rectification phenomenon in 3-Layer nanopores with varying interlayer spacings. (a) Schematic diagram of increasing the interlayer spacing in multilayered nanopores. The (b) ionic current and (c) ICR ratio as a function of the electric field *E* for different layer spacing. (d) FESs of K^+^ as a function of the axial (*z*) and radial (*r*) directions for 3-Layer nanopore with *S* = 0.68 nm. The black dashed line represents the minimum free energy path for K^+^ transport in the ON-state, and the red/blue dashed lines represent the modified positions of COO^–^/NH_3_^+^. (e) Schematic diagram of K^+^ hydration evolution during discontinuous transmembrane transport for 3-Layer nanopores with extended interlayer spacing at ON-state, the green arrows represent K^+^ transport pathways.

## Conclusions

In summary, our atomistic MD simulations reveal how interlayer coupling—modulated through layer number and spacing—critically governs rectification performance. It is found that increasing layer numbers significantly attenuates ion fluxes (e.g., current drops from 146.6 nA in one-layer systems to 42.9 nA in four-layer systems at 4.0 V/nm), yet concurrently enhances the ICR ratio by 2 orders of magnitude (from 2.3 to 2000 at 3.5 V/nm) and shifts the peak rectification field (*E*_max_) to higher voltages (0.7 → 3.5 V/nm). These cooperative effects demonstrate that multilayered architectures powerfully optimize ON/OFF switching performance despite reducing absolute current. Consequently, optimal design requires balancing current magnitude against ICR ratio when selecting layer number and spacing for specific applications.

Crucially, our results provide molecular-level mechanistic insights into enhanced rectification performance. Spatial density distributions reveal distinct ion arrangements dependent on layer number and operational state: While 1-Layer nanopores show minimal ion enrichment, multilayered systems (e.g., 3-/4-Layer) exhibit significant accumulation with pronounced ON/OFF-state differences. Hydration numbers remain stable in 1-Layer pores but undergo complete dehydration–rehydration cycles in multilayered structures due to strong interlayer coupling. Importantly, free energy analysis reveals a directionally asymmetric free energy landscape and a layer-number-dependent entropy–enthalpy competition in ion–pore interactions. Enhanced interlayer coupling with increasing layer number effectively modulates changes in Δ*G*, including the positions of energy barriers and wells. In particular, our work suggests that entropy—different from its role in selectivity^26–29^ —critically stabilizes energy wells in multilayered Janus pores to facilitate ion migration. The resulting entropy-regulated modulation of the free energy pathway provides a distinct thermodynamic basis for asymmetric ion transport and current rectification. Moreover, it also provides practical guidance for designing ionic rectification devices by making full use of the interlayer coupling effect—for instance, through precise adjustment of interlayer spacing. Collectively, these results elucidate how interlayer coupling governs ion dynamics and enhances rectification in multilayered Janus nanopores, providing essential theoretical guidance for designing high-performance rectified membranes and nanofluidic diodes.

## Supplementary Material



## Notes

Detailed methods for simulation and thermodynamic calculations; and supporting figures showing schematic diagrams, ion hydration numbers, concentration profiles, effects of pore size/shape and structural imperfections, layer-dependent transport properties, comparison with h-BN nanopores, free energy landscapes for anions, and influence of interlayer spacing on ion flux and hydration (PDF)
