# Supplementary material for: Harnessing Entropic Effects from Interlayer Coupling to Modulate Ion Transport and Rectification in Multilayered Janus Graphene Nanopores
Source: J Am Chem Soc. 2026 Jan 9;148(3):3240–9. doi: 10.1021/jacs.5c17242 (PMC12856899; doi:10.1021/jacs.5c17242)
Supplement: Supplementary file 1 [file ja5c17242_si_001.pdf]

Supporting Information

Harnessing Entropic Effects from Interlayer Coupling to Modulate Ion Transport and Rectification in Multilayered Janus Graphene Nanopores

Shuang Li,[1] Xinke Zhang,[1,*] Xuewei Dong,[1] Xin You,[1] Bing Yuan[2,*] and Kai Yang[1,*]

[1]Center for Soft Condensed Matter Physics and Interdisciplinary Research & School of Physical Science and Technology, Soochow University, Suzhou 215006, Jiangsu, China

[2]Songshan Lake Materials Laboratory, Dongguan 523808, Guangdong, China

*Corresponding authors. xkzhang@suda.edu.cn (X.Z.); yuanbing@sslab.org.cn (B.Y.); yangkai@suda.edu.cn (K.Y.)

Table of contents

Experimental Section (Simulation Parameters and Models and Calculations of Free Energy, Entropy, and Enthalpy).

Figure S1. Schematic diagram of a multilayered Janus nanopores with varying layer numbers and interlayer spacing.

Figure S2. The ions hydration number for 3-Layer nanopore under different electric field strengths.

Figure S3. Ion concentration of ions versus z-position at ±*E* varies with electric field strength.

Figure S4. Effect of pore size on rectification performance and the associated thermodynamic mechanism of ion transport.

Figure S5. Effect of pore shape on rectification performance and the associated thermodynamic mechanism of ion transport.

Figure S6. Effect of structural imperfection (random interlayer spacing and misaligned layers) on rectification performance and the associated thermodynamic mechanism of ion transport.

Figure S7. Ionic current, ICR ratio, and ion−sheet interaction energy as functions of the layer number.

Figure S8. Comparison of rectification performances of GO and h-BN nanopores and the associated ion transport thermodynamic mechanisms.

Figure S9. ON-state and OFF-state densities for 2-/4-Layer nanopores.

Figure S10. ON-state ions hydration number for 2-/4-Layer nanopores.

Figure S11. OFF-state ion hydration number for 1 to 4 layered nanopores

Figure S12. The free energy surfaces as well as the changes in free energy, entropy, and enthalpy along the *z*-axis of the nanopores for Cl^-.

Figure S13. In 3-Layer nanopore, the ion flux for system with distinct layer spacing.

Figure S14. ON-state ions hydration number for different layer spacing.

EXPERIMENTAL SECTION

Simulation Parameters and Models. All molecular dynamics simulations were performed using GROMACS 2024.[1] The system was maintained at 300 K via the Nosé-Hoover thermostat.[2] We employed the AMBER03 force field for graphene carbon atoms and charged residues,[3] with ion parameters adopted from the validated studies.[4] Water molecules were modeled using the SPC/E model,[5] which can accurately reproduce experimental diffusion properties. Electrostatic interactions were calculated via the Particle Mesh Ewald (PME) method (real-space cutoff: 1.2 nm), with van der Waals interactions truncated at 1.2 nm.[6] Periodic boundary conditions were applied in all dimensions, similar to previous works.[7-9] The graphene sheets were position-restrained while pore-edge residues remained flexible. All simulations comprised an initial 5 ns equilibration period followed by a 120 ns production phase for data acquisition. Trajectories were sampled every 1 ps using a 2.5 fs integration time step. To ensure the computational convergence, two independent simulation replicas were performed for each system.

For h-BN System, partial charges of Q_B = +0.4e for boron and Q_N = –0.4e for nitrogen were assigned based on optimized monolayer parameters.[10-11] The CHARMM36 force field was used for boron and nitrogen atoms.[12] All remaining simulation parameters matched those of the GO systems. Positively or negatively charged pore edges were created by selectively removing B or N atoms, yielding h-BN nanopores with geometries analogous to the GO nanopores described in the main text. Electrically distinct h-BN sheets were then stacked to form Janus nanopores.

Calculations of Free Energy, Entropy, and Enthalpy. The free energy surfaces (FESs) for ion transport along the axial (z) and radial (r) coordinates were calculated using On-the-fly Probability Enhanced Sampling (OPES) as implemented in the PLUMED 2.9.1 plugin.[13-14] This approach could provide the corresponding free energy changes (ΔG) along the translocation axis (i.e., ions migration direction) and allow identification of the minimum free energy pathways for ions traversing the nanopores. Furthermore, we decomposed ΔG into entropy and enthalpy contributions based on the

Gibbs free energy equation using the finite-difference approximations: $-T\Delta S = T\frac{\mathrm{d}G}{\mathrm{d}T} \approx \frac{T}{2\Delta T}(G(T+\Delta T) - G(T-\Delta T))$ and $\Delta H = \Delta G - (-T\Delta S)$, as established in previous stuied.[7-8, 15-17] All OPES calculations for ΔG were performed at three temperatures (295 K, 300 K, and 305 K) to enable the thermodynamic decomposition.

In addition, the contributions of hydration entropy and ion–ion configurational entropy were also calculated. To estimate ionic hydration entropy (ΔS_h), we quantified the variation in the first hydration shell during the transport of a single K^+.[18] Specifically, we computed the angular distribution $P(\theta,\varphi;z)$ of water molecules in the first hydration shell relative to the K^+–O direction and compared it with the reference bulk distribution $P_\mathrm{bulk}(\theta,\varphi)$. The hydration entropy change ΔS_h was then estimated using a Kullback–Leibler divergence-like orientational entropy formulation:

$$\Delta S_\mathrm{h} = -N(z)\mathrm{k_B}\sum P(\theta,\varphi;z)\ln\left(\frac{P(\theta,\varphi;z)}{P_\mathrm{bulk}(\theta,\varphi)}\right) + (N_0 - N(z))\Delta S_0,$$

where $N(z)$ and N_0 are the average numbers of coordinating water molecules at position z and in the bulk, respectively. The first term captures the orientational ordering of water molecules in the hydration shell: a strong deviation of $P(\theta,\varphi;z)$ from the bulk distribution reduces the hydration entropy. The second term accounts for partial dehydration, and each water molecule released from the ion to the bulk contributes an entropy gain of $\Delta S_0 \approx 1\ \mathrm{k_B}$.[19]

To calculate ion–ion configurational entropy (ΔS_c), we computed the relative orientation distribution $P(\theta,\varphi;z)$ of K^+–K^+ and K^+–Cl^- coordination pairs, using the bulk coordination distribution $P_0(\theta,\varphi)$ as a reference. The ion–ion configurational entropy change ΔS_c was calculated as:

$$\Delta S_\mathrm{c} = -N_\mathrm{pair}(z)\mathrm{k_B}\sum P(\theta,\varphi;z)\ln\left(\frac{P(\theta,\varphi;z)}{P_0(\theta,\varphi)}\right).$$

Here, N_Pair represents the average number of coordinating ions at position z. ΔS_c captures the change in configurational freedom within the local ionic coordination shell at each pore location. Furthermore, to facilitate comparison with the system entropy, each entropy term was scaled by the effective number of transported ions (N_eff), which was derived from the average ion occupancy within the nanopore region.

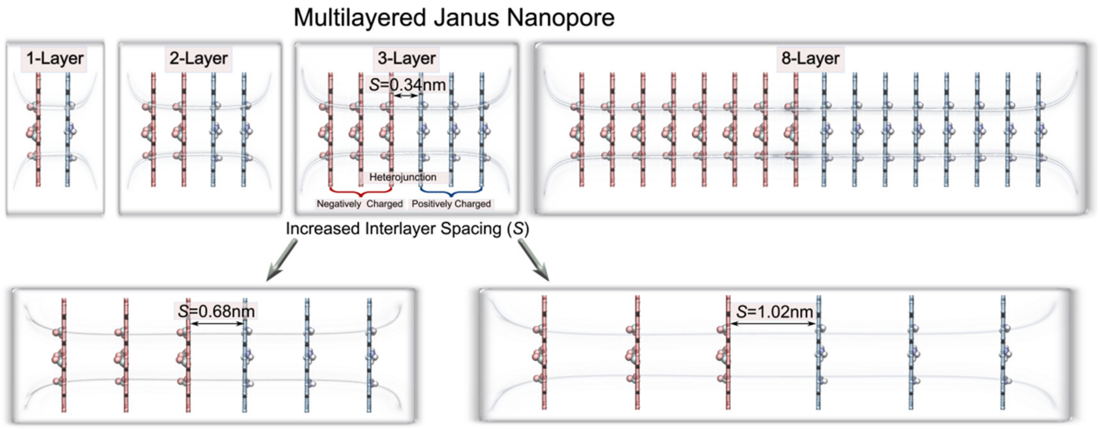

Figure S1. Schematic diagram of a multilayered Janus nanopores with varying layer numbers and interlayer spacing (*S*).

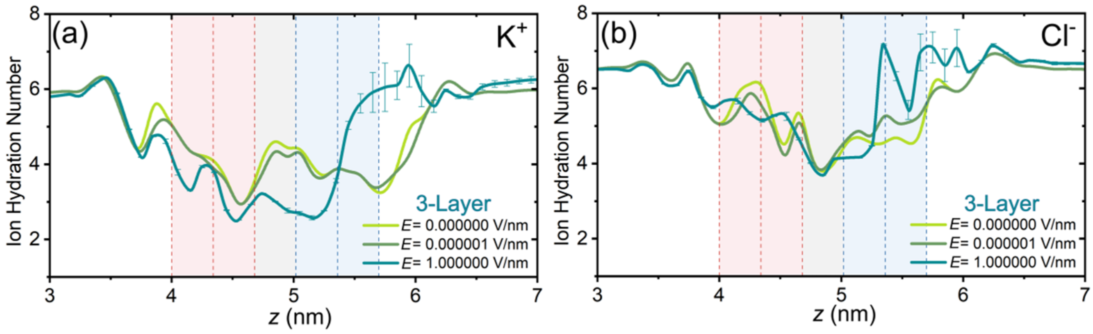

Figure S2. The hydration number of (a) K^+ and (b) Cl^- as a function of the position along *z*-axis for 3-Layer nanopore under different electric field strengths; dashed lines indicate COO^-/NH_3^+ modification sites. Light red/blue regions: positive/negative charged; gray: heterojunction.

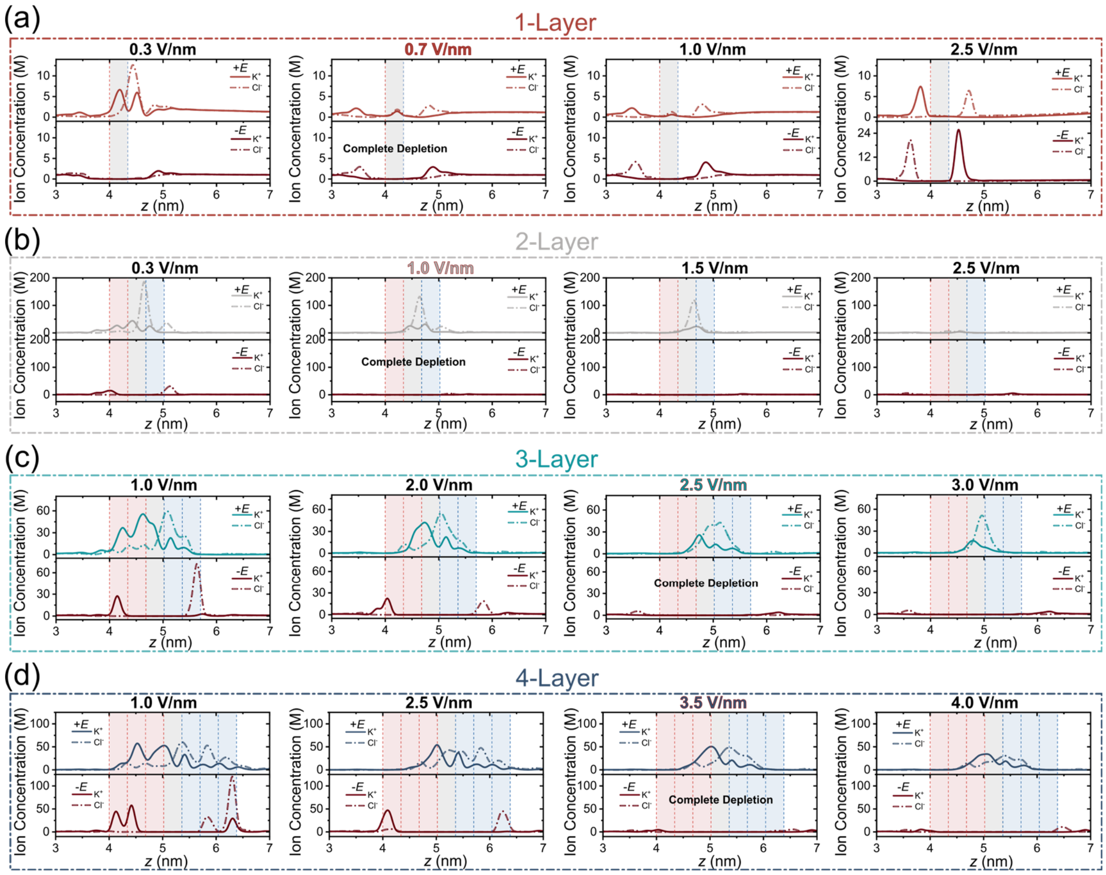

Figure S3. Ion concentration of ions versus z-position at ±*E* varies with electric field strength for (a) 1-Layer, (b) 2-Layer, (c) 3-Layer, and (d) 4-Layer nanopores. Dashed lines indicate COO^-/NH_3^+ modification sites; Light red/blue regions: positive/negative charged; gray: heterojunction. The electric field strength at which ions are completely depleted inside nanopore at −*E* is marked in color.

Note: The shift in the ionic rectification peak with layer number correlates directly with the evolution of ion concentration polarization (ICP) inside the Janus nanopores. This correlation is substantiated by calculating the axial (*z*) ion concentration profiles for nanopores of different layer numbers under various applied fields (**Figure S3**).

In general, ions preferentially accumulate in regions of opposite charge due to electrostatic attraction, and this redistribution strongly depends on the field direction. Under +*E*, ions enter the nanopore from the oppositely charged entrance, making the pore interior an ion-enriched region. In contrast, under −*E*, ions struggle to enter from the like-charged entrance, causing the nanopore to become an ion-depleted region. This field-dependent ICP contrast is the key factor that drives ion current rectification in

Janus nanopores, and the behavior of ICP varies noticeably with both the pore geometry and the applied field strength. For the 1-Layer nanopore, the ion concentration in the pore region declines steadily as the field increases, and a fully depleted state appears at approximately –0.7 V/nm. In contrast, multilayered nanopores exhibit more pronounced ion trapping due to interlayer coupling, which produces deeper free energy wells. As a result, a stronger negative field is needed for the complete depletion. Notably, complete depletion and the ICR maximum occur at the same field. This correspondence indicates the essential role of ICP in rectification devices, which is dominated by the interlayer coupling.

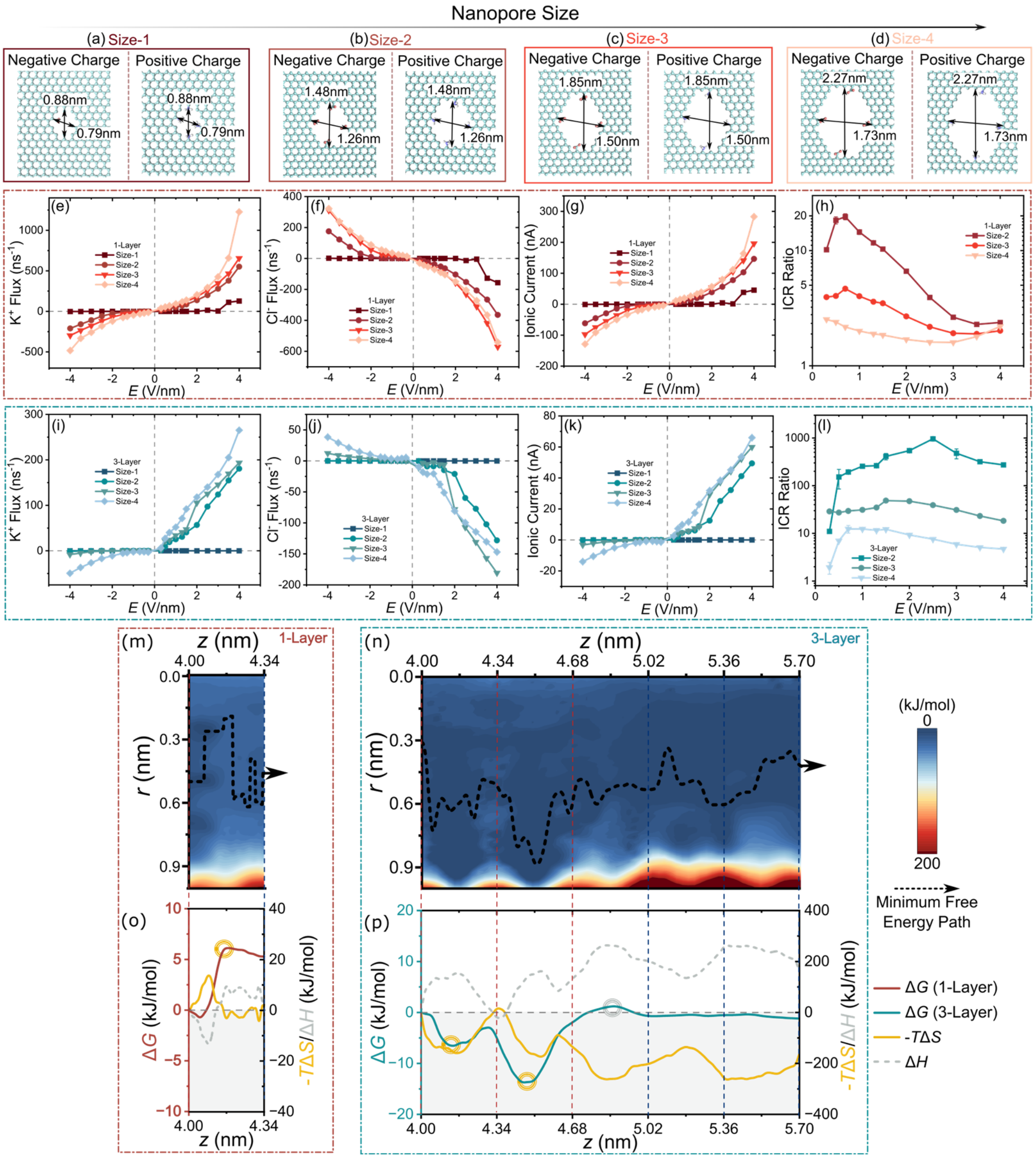

Figure S4. Effect of pore size on rectification performance and the associated thermodynamic mechanism of ion transport. (a–d) GO nanopores of different sizes. (e–h) Rectification performance of the 1-Layer pore: (e) K^+ flux profile, (f) Cl^- flux profile, (g) ionic current, and (h) ICR ratio as a function of electric field E for each pore size. (i–l) Rectification performance of the 3-Layer pore: (i) K^+ flux profile, (j) Cl^- flux profile, (k) ionic current, and (l) ICR ratio as a function of electric field E for each pore size. (m–n) free energy surfaces (FESs) of K^+ as a function of axial (z) and radial (r) coordinates in a nanopore with size-4: (m) 1-Layer; (n) 3-Layer. The black dashed line marks the minimum free energy path for K^+ transport in the ON-state. The changes in free energy (ΔG), entropy (ΔS), and enthalpy (ΔH) of K^+ along the z-axis for (o) 1-Layer nanopore and (p) 3-Layer nanopore. Red/blue dashed lines indicate the modified positions of COO^-/NH_3^+ groups. In (o) and (p), circles highlight peaks and valleys of the free energy barrier (yellow: entropy-dominated; gray: enthalpy-dominated).

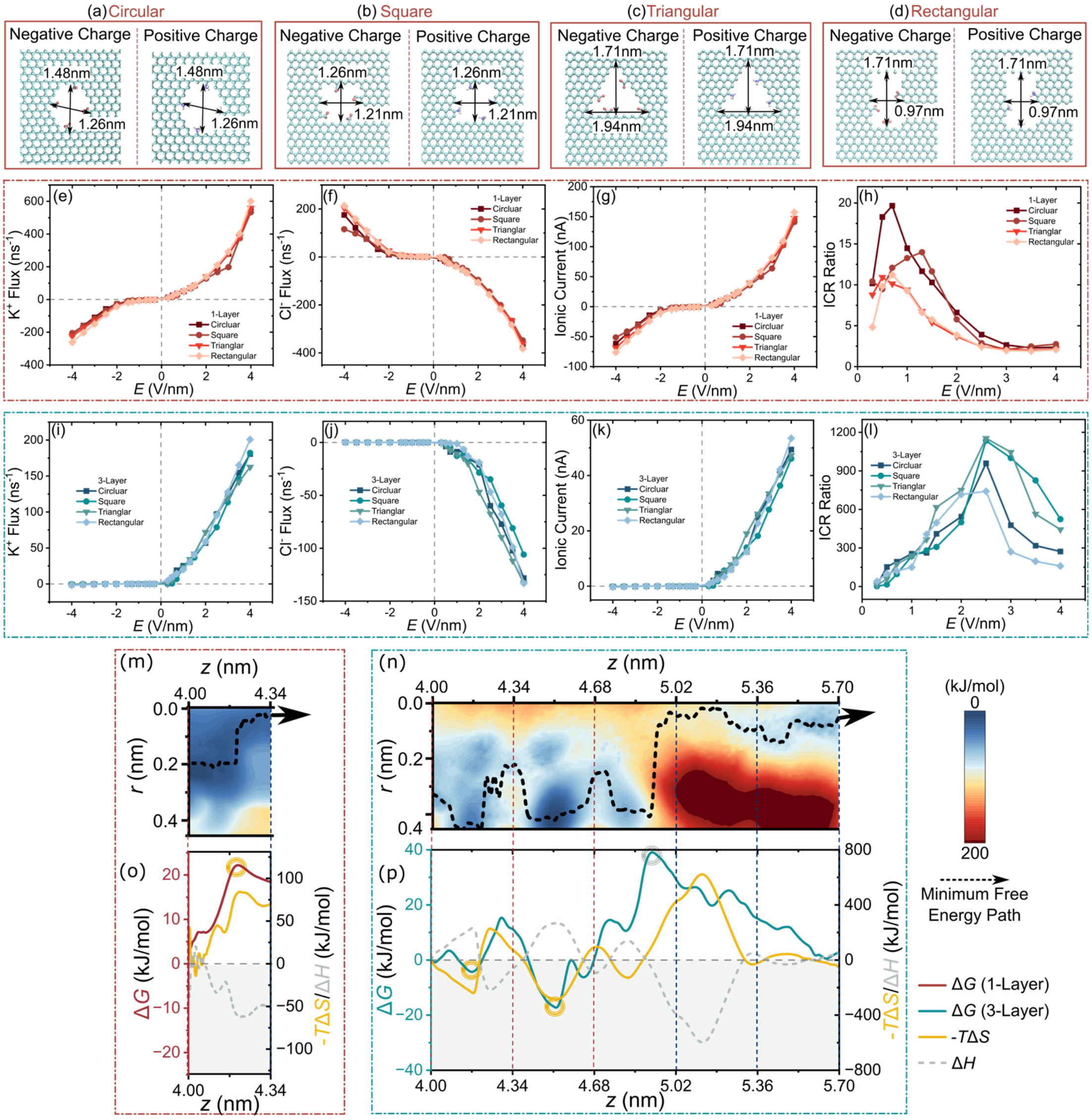

Figure S5. Effect of pore shape on rectification performance and the associated thermodynamic mechanism of ion transport. (a–d) GO nanopores of different shapes. (e–h) Rectification performance of the 1-Layer pore: (e) K^+ flux profile, (f) Cl^- flux profile, (g) ionic current, and (h) ICR ratio as a function of electric field E for each pore shape. (i–l) Rectification performance of the 3-Layer pore: (i) K^+ flux profile, (j) Cl^- flux profile, (k) ionic current, and (l) ICR ratio as a function of electric field E for each pore shape. (m–n) free energy surfaces (FESs) of K^+ as a function of axial (z) and radial (r) coordinates in a square nanopore: (m) 1-Layer; (n) 3-Layer. The black dashed line marks the minimum free energy path for K^+ transport in the ON-state. The changes in free energy (ΔG), entropy (ΔS), and enthalpy (ΔH) of K^+ along the z-axis for (o) 1-Layer nanopore and (p) 3-Layer nanopore. Red/blue dashed lines indicate the modified positions of COO^-/NH_3^+ groups. In (o) and (p), circles highlight peaks and valleys of the free energy barrier (yellow: entropy-dominated; gray: enthalpy-dominated).

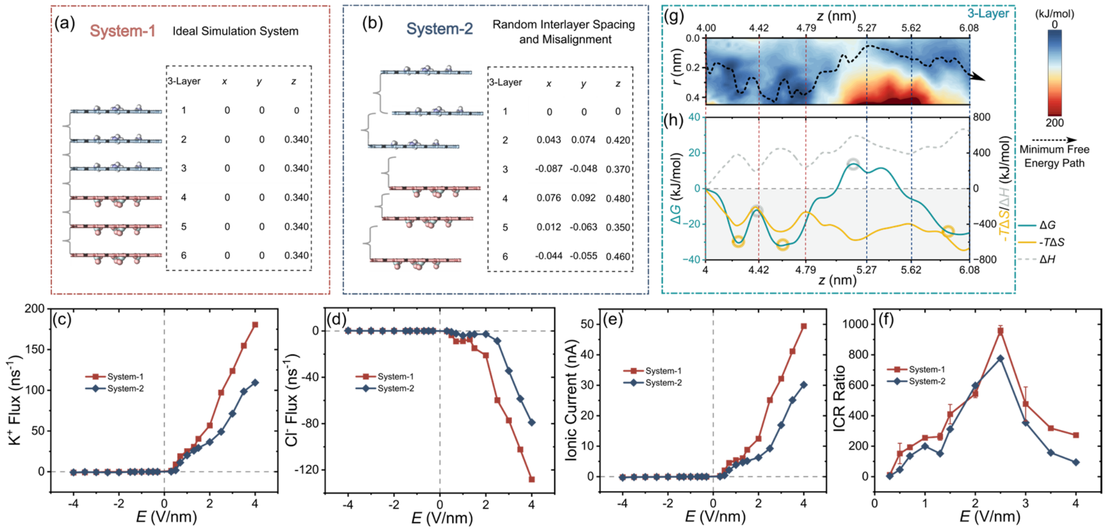

Figure S6. Effect of structural imperfection (random interlayer spacing and misaligned layers) on rectification performance and the associated thermodynamic mechanism of ion transport. (a–b) GO nanopores with or without structural imperfection. (c–f) Comparison of rectification performance. (g–h) Free energy surfaces (FESs) of K^+ as a function of axial (*z*) and radial (*r*) coordinates. The black dashed line marks the minimum free energy path for K^+ transport in the ON-state. Red/blue dashed lines indicate the modified positions of COO^-/NH_3^+ groups. In (h), circles highlight peaks and valleys of the free energy barrier (yellow: entropy-dominated; gray: enthalpy-dominated).

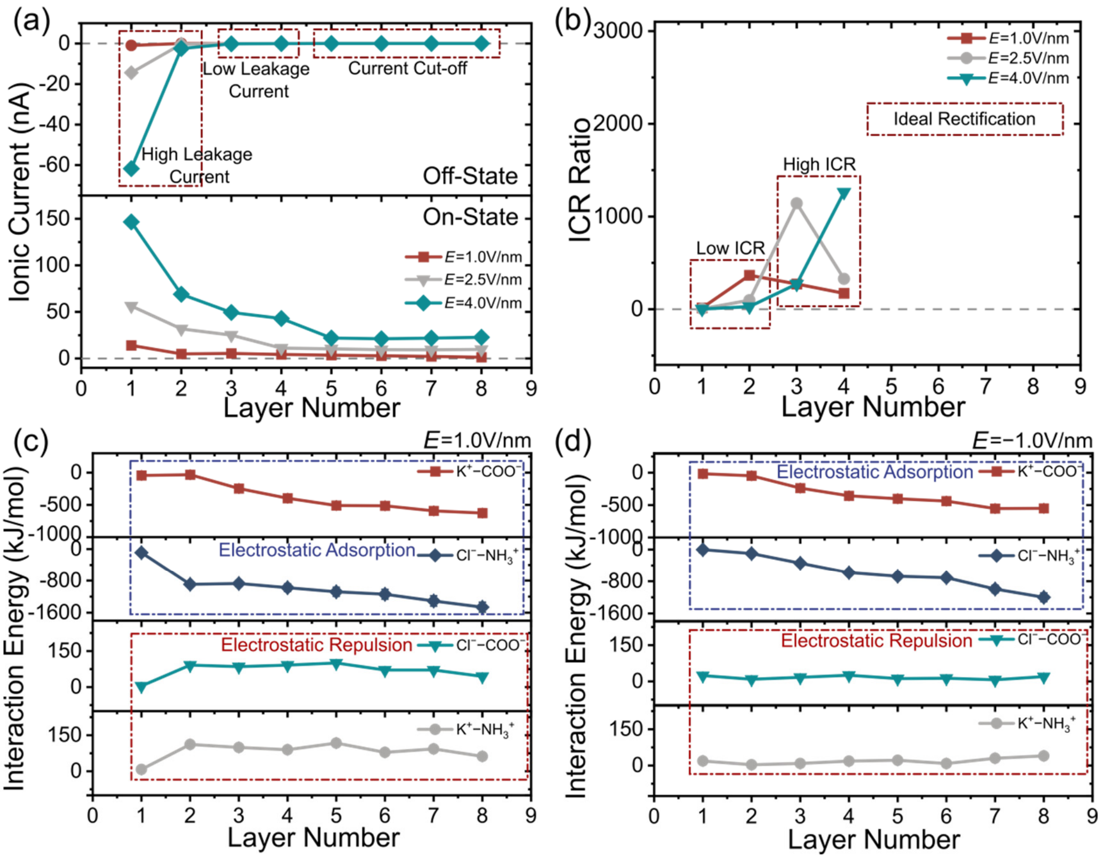

Figure S7. Ionic current, ICR ratio, and ion-sheet interaction energy as functions of the layer number. (a) Ionic current through Janus nanopores with varying layer numbers (ranging from 1-Layer to 8-Layer) under representative electric field strengths (E). (b) Corresponding ICR ratios. (c, d) Mean interaction energies between ions and individual sheets (including the $-NH_3^+$ sheet and $-COO^-$ sheet) in Janus nanopores with different layer configurations, under representative electric field strengths (±1.0 V/nm).

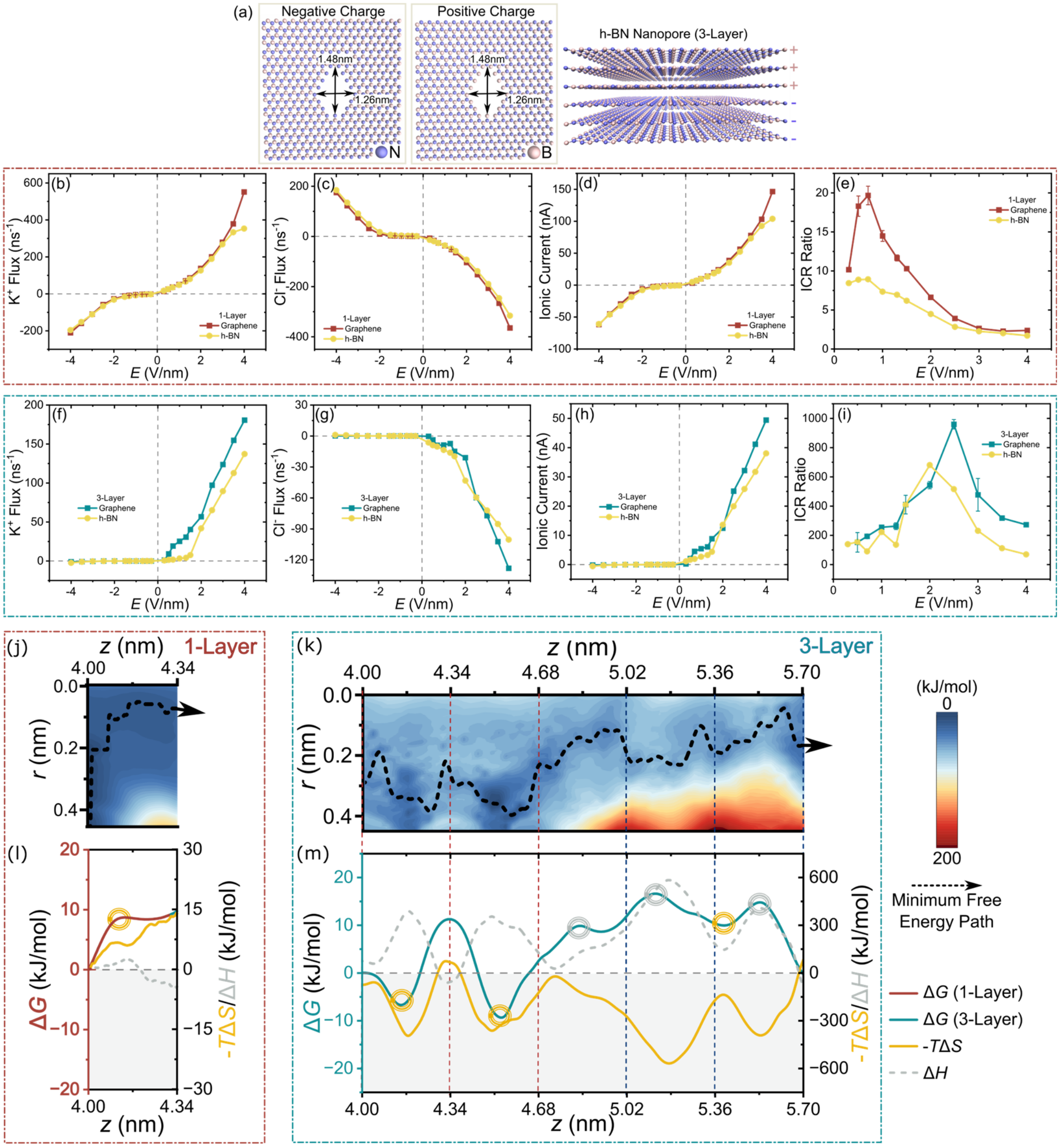

Figure S8. (a) Schematic diagram of the Janus h-BN nanopore. (b–i) Comparison of rectification performance between h-BN and GO nanopores: (b–e) 1-Layer pores; (f–i) 3-Layer pores. (j–k) Free energy surfaces (FESs) of K^+ as a function of axial (z) and radial (r) coordinates in h-BN nanopore: (j) 1-Layer; (k) 3-Layer. The black dashed line marks the minimum free energy path for K^+ transport in the ON-state. The changes in free energy (ΔG), entropy (ΔS), and enthalpy (ΔH) of K^+ along the z-axis for (l) 1-Layer nanopore and (m) 3-Layer nanopore. Red/blue dashed lines indicate the modified positions of COO^-/NH_3^+ groups. In (l) and (m), circles highlight peaks and valleys of the free energy barrier (yellow: entropy-dominated; gray: enthalpy-dominated).

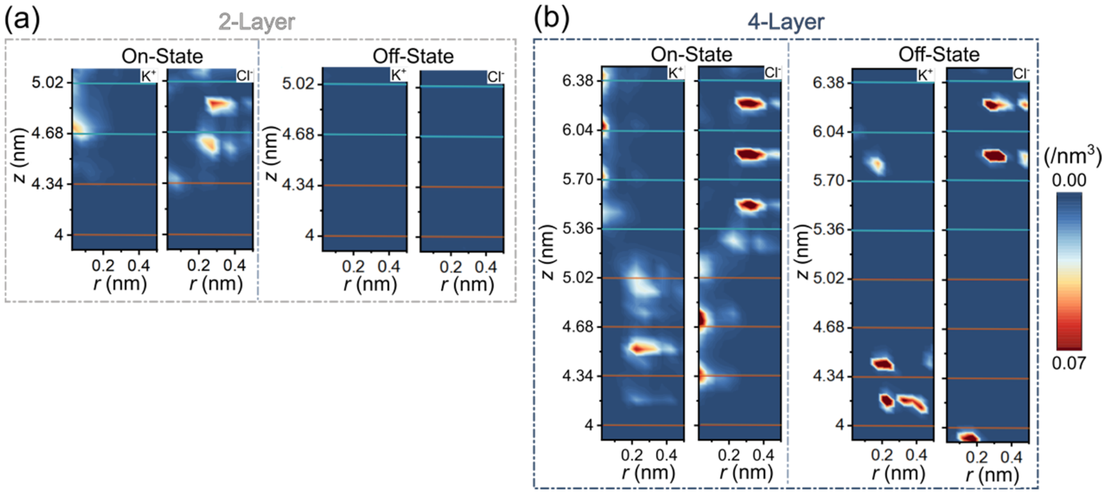

Figure S9. (a, b) ON-state (E = +1.0 V/nm) and OFF-state (E = −1.0 V/nm) densities for 2-/4-Layer nanopores. Horizontal lines denote COO^- (red)/NH_3^+ (blue) modification sites.

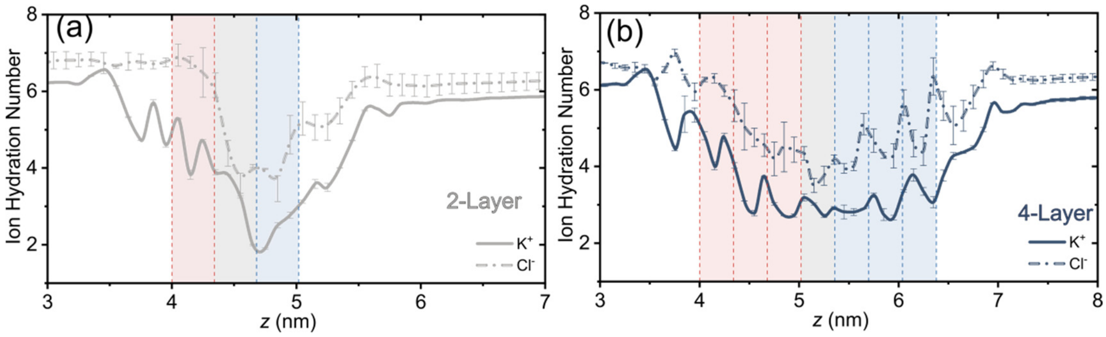

Figure S10. At ON-state (E = 1.0 V/nm), the ions hydration number as a function of the position along z-axis for (a) 2-Layer and (b) 4-Layer nanopore; dashed lines indicate COO^-/NH_3^+ modification sites. Light red/blue regions: positive/negative charged; gray: heterojunction.

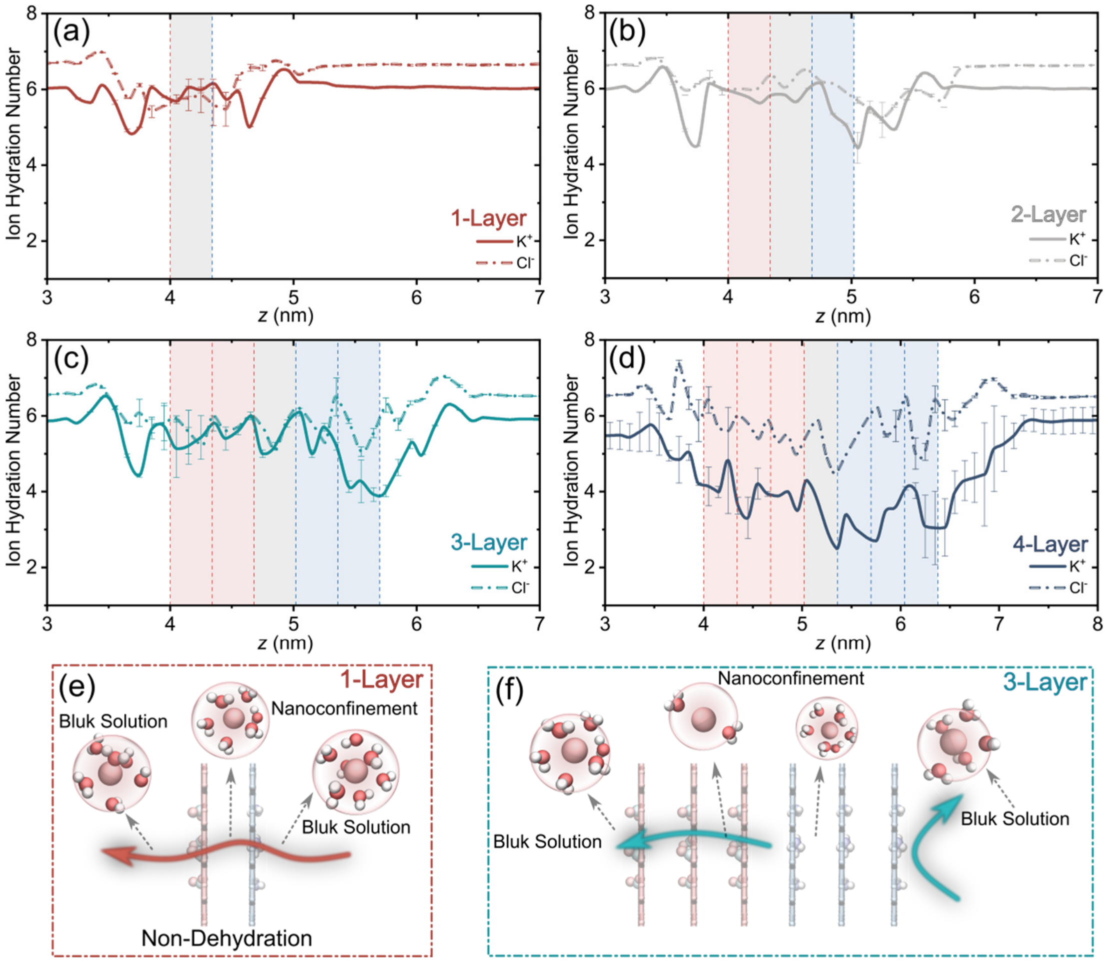

Figure S11. At OFF-state (E = –1.0 V/nm), the ion hydration number as a function of the position along z-axis for (a—d) 1 to 4 layered nanopores; dashed lines indicate COO^{-}/NH_3^{+} modification sites. Light red/blue regions: positive/negative charged; gray: heterojunction. Schematic Diagram of K^+ hydration evolution during transmembrane transport for (e) 1-Layer and (f) 3-Layer nanopores at OFF-state, the red/green arrows represent K^+ transport pathways.

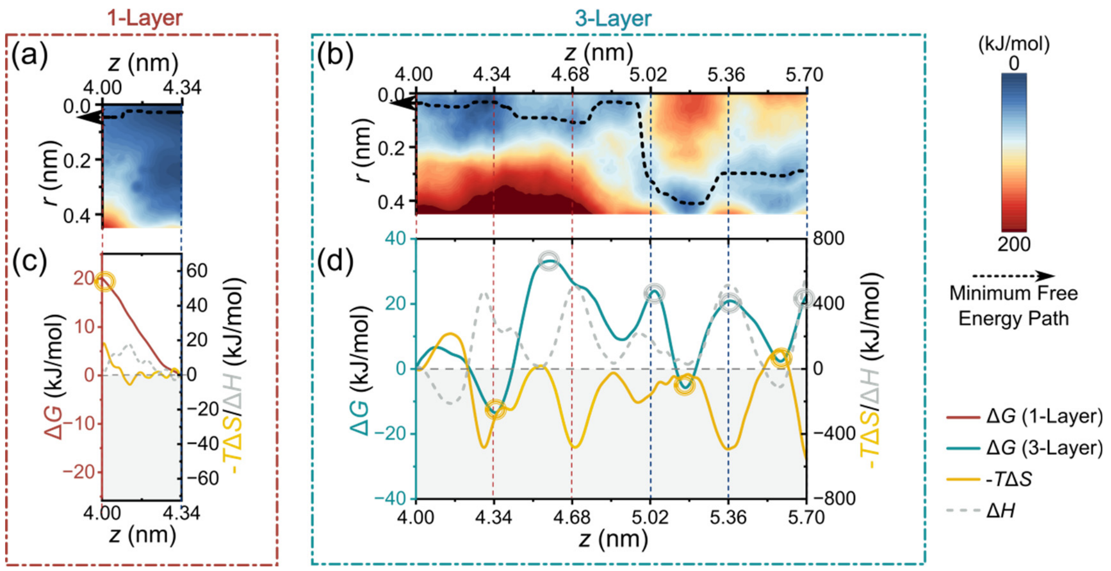

Figure S12. The free energy surfaces (FESs) during ion transport, as well as the changes in free energy, entropy, and enthalpy along the *z*-axis of the nanopores. FESs of Cl^- as a function of the axial (*z*) and radial (*r*) directions for (a) 1-Layer nanopore and (b) 3-Layer nanopore. The black dashed line represents the minimum free energy path for Cl^- transport in the ON-state. The changes in free energy, entropy, and enthalpy of Cl^- along the *z*-axis for (c) 1-Layer nanopore and (d) 3-Layer nanopore. The red/blue dashed lines represent the modified positions of COO^-/NH_3^+. The circles in Figures S12c,d represent the peaks and valleys of the free energy barrier (yellow: entropy dominance; gray: enthalpy dominance).

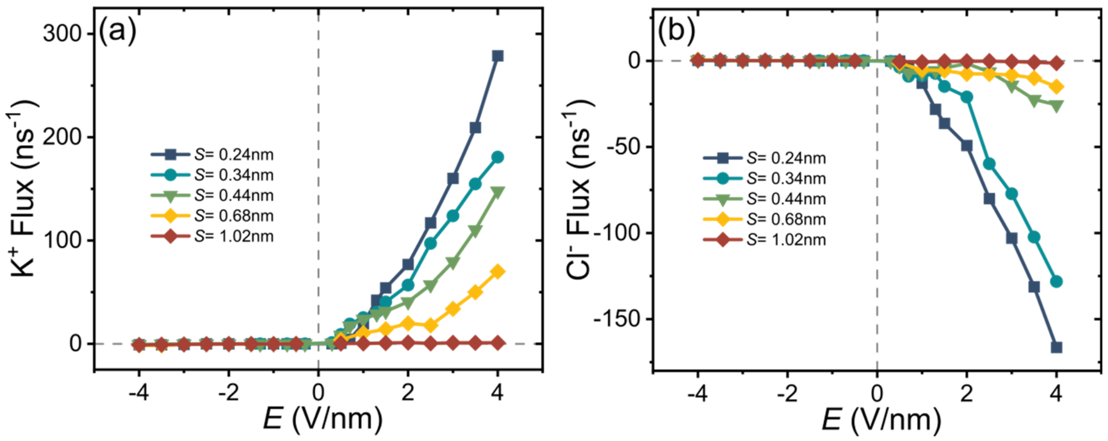

Figure S13. In 3-Layer nanopore, the flux profiles of (b) K^+ and (c) Cl^-versus electric field E for system with distinct layer spacing.

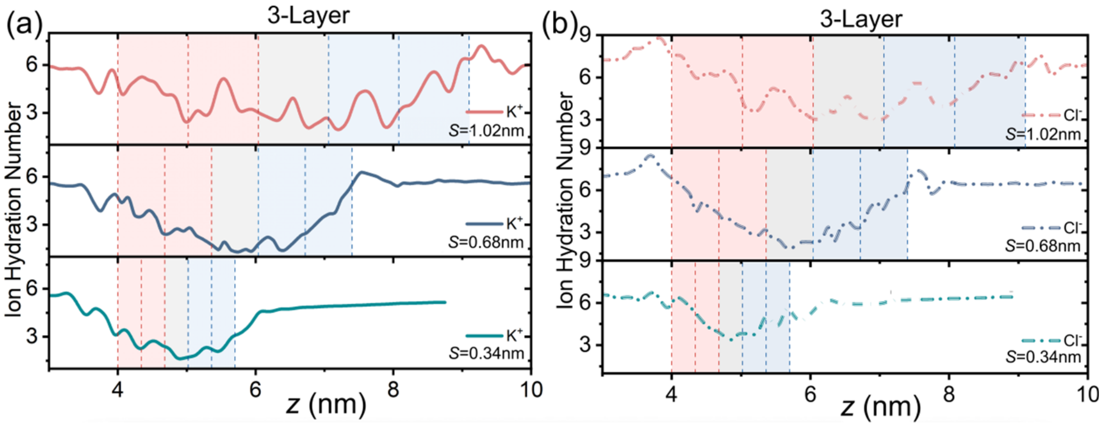

Figure S14. At E = 1.0 V/nm for ON-state, the hydration number as a function of the position along z-axis for (d) K^+ and (e) Cl^- for different layer spacing; dashed lines indicate COO^-/NH_3^+ modification sites. Light red/blue regions: positive/negative charged; gray: heterojunction.

■ REFERENCES

(1) Hess, B.; Kutzner, C.; van der Spoel, D.; Lindahl, E. GROMACS 4: Algorithms for Highly Efficient, Load-Balanced, and Scalable Molecular Simulation. *J. Chem. Theory Comput.* **2008,** *4* (3), 435–447.

(2) Nose, S.; Klein, M. L. Constant Pressure Molecular Dynamics for Molecular Systems. *Mol. Phys.* **1983,** *50* (5), 1055–1076.

(3) Duan, Y.; Wu, C.; Chowdhury, S.; Lee, M. C.; Xiong, G. M.; Zhang, W.; Yang, R.; Cieplak, P.; Luo, R.; Lee, T.; Caldwell, J.; Wang, J. M.; Kollman, P. A Point-Charge Force Field for Molecular Mechanics Simulations of Proteins Based on Condensed-Phase Quantum Mechanical Calculations. *J. Comput. Chem.* **2003,** *24* (16), 1999–2012.

(4) Chowdhuri, S.; Chandra, A. Hydration Structure and Diffusion of Ions in Supercooled Water: Ion Size Effects. *J. Chem. Phys.* **2003,** *118* (21), 9719–9725.

(5) Salman, S.; Zhao, Y. Z.; Zhang, X. K.; Su, J. Y. Effect of Temperature on the Coupling Transport of Water and Ions through a Carbon Nanotube in an Electric Field. *J. Chem. Phys.* **2020,** *153* (18), 184503.

(6) Essmann, U.; Perera, L.; Berkowitz, M. L.; Darden, T.; Hsing, L.; Pedersen, L. G. A Smooth Particle Mesh Ewald Method. *J. Chem. Phys.* **1995,** *103* (19), 8577–8593.

(7) Ou, L.; Chen, H.; Yuan, B.; Yang, K. Membrane-Specific Binding of 4 nm Lipid Nanoparticles Mediated by an Entropy-Driven Interaction Mechanism. *ACS Nano* **2022,** *16* (11), 18090–18100.

(8) You, X.; Dong, X. W.; Tu, W. Q.; Yuan, B.; Yang, K. Conformational Versus Configurational Entropy: Deciphering the Alkyl Chain-Dependent Membrane Attack Mechanism of Ionic Liquid Derivatives. *ACS Nano* **2025**, *19* (30), 27930–27940.

(9) Niu, J.; Dong, X.; Pan, W.; Yuan, B.; Yang, K. Molecular Insights into the Membrane Phase Separation Influenced by Membrane/Lipid Structural Changes. *Chem. Res. Chin. Univ.* **2025,** DOI: 10.1007/s40242-025-5083-z

(10) Won, C. Y.; Aluru, N. R. Structure and Dynamics of Water Confined in a Boron Nitride Nanotube. *J. Phys. Chem. C* **2008,** *112* (6), 1812–1818.

(11) Luan, B. Q.; Zhou, R. H., Atomic-Scale Fluidic Diodes Based on Triangular

Nanopores in Bilayer Hexagonal Boron Nitride. *Nano Lett.* **2019,** *19* (2), 977–982.

(12) Bjelkmar, P.; Larsson, P.; Cuendet, M. A.; Hess, B.; Lindahl, E. Implementation of the CHARMM Force Field in GROMACS: Analysis of Protein Stability Effects from Correction Maps, Virtual Interaction Sites, and Water Models. *J. Chem. Theory Comput.* **2010,** *6* (2), 459–466.

(13) Bussi, G.; Laio, A. Using Metadynamics to Explore Complex Free-Energy Landscapes. *Nat. Rev. Phys.* **2020,** *2* (4), 200–212.

(14) Bonomi, M.; Barducci, A.; Parrinello, M. Reconstructing the Equilibrium Boltzmann Distribution from Well-Tempered Metadynamics. *J. Comput. Chem.* **2009,** *30* (11), 1615–1621.

(15) Wei, L.; Tu, W. Q.; Xu, Y. W.; Xu, C.; Dou, Y. J.; Ge, Y. K.; Sun, S. Q.; Wei, Y. S.; Yang, K.; Yuan, B. Assembly-Induced Membrane Selectivity of Artificial Model Peptides through Entropy-Enthalpy Competition. *ACS Nano* **2024,** *18* (28), 18650–18662.

(16) MacCallum, J. L.; Tieleman, D. P. Computer Simulation of the Distribution of Hexane in a Lipid Bilayer: Spatially Resolved Free Energy, Entropy, and Enthalpy Profiles. *J. Am. Chem. Soc.* **2006,** *128* (1), 125–130.

(17) Liu, G. Q.; Xu, Z. Y.; Dai, X. B.; Zeng, Y.; Wei, Y.; He, X. Z.; Yan, L. T.; Tao, L., *De Novo* Design of Entropy-Driven Polymers Resistant to Bacterial Attachment via Multicomponent Reactions. *J. Am. Chem. Soc.* **2021,** *143* (41), 17250–17260.

(18) Noh, Y.; Riccardi, D.; Smolyanitsky, A. Entropic Modulation of Divalent Cation Transport. *Phys. Rev. Lett.* **2025,** *135* (13), 138001.

(19) Florián, J.; Warshel, A. Calculations of Hydration Entropies of Hydrophobic, Polar, and Ionic Solutes in the Framework of the Langevin Dipoles Solvation Model. *J. Phys. Chem. B* **1999,** *103* (46), 10282–10288.
